# *GmHs1-1* and *GmqHS1* Simultaneously Contribute to the Domestication of Soybean Hard-Seededness

**DOI:** 10.3390/plants13152061

**Published:** 2024-07-26

**Authors:** Huifang Yan, Daicai Tian, Qian Zhang, Jiangqi Wen, Zeng-Yu Wang, Maofeng Chai

**Affiliations:** 1 Key Laboratory of National Forestry and Grassland Administration on Grassland Resources and Ecology in the Yellow River Delta, College of Grassland Science, Qingdao Agricultural University, Qingdao 266109, China; 2 Institute for Agricultural Biosciences, Oklahoma State University, Ardmore, OK 73401, USA; 3 Noble Research Institute, Ardmore, OK 73401, USA

**Keywords:** soybean, hard-seededness, domestication

## Abstract

Seed physical dormancy (hard-seededness) is an interesting ecological phenomenon and important agronomic trait. The loss of seed coat impermeability/hard-seededness is a key target trait during the domestication of leguminous crops which allows seeds to germinate rapidly and uniformly. In this study, we examined the mutation of quantitative trait locus (QTL) genes, *GmHs1-1* and *GmqHS1*, in 18 wild soybean (*G. soja*) and 23 cultivated soybean (*G. max*) accessions. The sequencing results indicate that a G-to-T substitution in *GmqHS1* and a C-to-T substitution in *GmHs1-1* occurred in all 23 cultivated soybean accessions but not in any of the 18 wild soybean accessions. The mutations in the two genes led to increased seed coat permeability in cultivated soybean. Therefore, we provide evidence that two genes, *GmHs1-1* and *GmqHS1*, simultaneously contribute to the domestication of hard-seededness in soybeans. This finding is of great significance for genetic analysis and improved utilization of the soybean hard-seededness trait.

## 1. Introduction

Hard-seededness is an important trait related to soybean domestication. Compared with non-hard seeds, hard soybean seeds have higher seed vigor and longer seed lifespan [1,2,3]. Additionally, hard seeds are less prone to imbibition, delaying seed deterioration and facilitating seed preservation and transportation [2]. However, hard-seededness is not convenient for seed production in agriculture and utilization in daily life, as it not only reduces field emergence and final yield, but also affects seed consumption and processing. The breakdown of seed coat impermeability/hard-seededness is a crucial step in soybean domestication and improvement to produce high-value cultivated seeds [4]. Loss of seed coat impermeability is a key trait for many leguminous crops, which allows seeds to germinate rapidly and uniformly [5,6,7]. Permeable seed coats are also beneficial in processing seeds to produce vegetable oil and soy foods [8].

As we were interested in the seed coat permeability phenotype of legume species, two papers published in 2015 attracted our attention with their interesting results on soybean. Sun et al. [9] reported that seed coat impermeability in soybean is controlled by a major quantitative trait locus (QTL) gene, *GmHs1-1*, encoding a calcineurin-like protein. Soon after, another major QTL gene, *qHS1*, was also identified to control seed coat impermeability in soybean by fine mapping [10]. *qHS1*, an endo-1, 4-β-glucanase gene, promotes the accumulation of 1, 4-β-glucan in the outer layer of palisade cells, leading to the production of hard seeds. Moreover, *qHS1* had the greatest effect on soybean impermeability according to another study [11]. Interestingly, the locations of these two genes are close to each other on the physical map. It is possible that these two genes function together to improve the seed coat permeability in cultivated soybean (*Glycine max*) [12], but each set of authors missed the gene identified by the other during their fine-mapping processes. To test the above hypothesis, we analyzed the single-nucleotide polymorphism (SNP) variation in different wild soybean (*Glycine soja*) and elite cultivated soybean populations. Our findings, unsurprisingly, indicated that these two genes, not one, simultaneously exist and contribute to improving seed coat permeability in cultivated soybean. Our analyses suggest that both the *Hs1-1* and *qHS1* genes are functional in controlling seed coat impermeability in legume species. *Hs1-1* and *qHS1* function together to facilitate cultivated soybean domestication for seed coat permeability.

## 2. Results and Discussion

**Figure 1:**
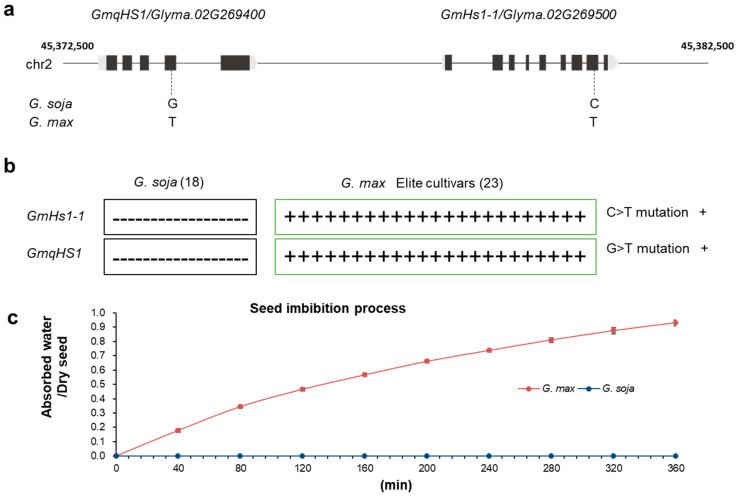
Single-nucleotide polymorphism locations in *G. soja* and *G. max*. (**a**) Physical map of *GmqHS1* and *GmHs1-1* in the soybean genome from phytozome and the key SNP variations controlling seed coat impermeability. The physical distance between *GmqHS1* and *GmHs1-1* is 4175 bp. (**b**) Distribution of SNP mutations in *GmHs1-1* and *GmqHS1* among 18 different *G. soja* and 23 *G. max* populations. (**c**) Typical imbibition process of *G. soja* and *G. max* seeds.

Hard-seededness is one of the crucial target traits during domestication of many legume crops [5,7,11]. Modern cultivated soybean, the most economically important legume crop, is commonly believed to have been domesticated from wild soybean in East Asia about 5000 years ago [13,14]. Previous studies indicated that several genes/QTLs control the hard-seededness [11]. Keim et al. [15] first used five RFLP markers to detect the QTLs of soybean hard-seededness, which were located on chromosomes 2, 3, 8 and 19, and explained 71.0% of the genetic variation. Watanabe et al. [16] identified three markers (*RAS1-3*) related to soybean hard-seededness and found that the dominant QTL (*RAS2*) was located on chromosome 2. In soybean PI594619, a single gene located between the markers Sat_202 and Satt459 on chromosome 2 was also identified to control seed coat impermeability [17]. Subsequently, two genes, *qHS1* and *GmHs1-1*, related to soybean hard-seededness on chromosome 2 were cloned independently [9,10]. It is possible that the QTLs on chromosome 2 in soybean have the greatest effect on seed coat impermeability [15,18]. Interestingly, according to current research information, *GmHs1-1* and *GmqHS1* were mapped close to each other on chromosome 2 in the soybean genome (Figure 1a) by two different independent research groups [9,10].

Investigating the relationship between these two genes in cultivated soybean varieties will help us clearly understand the trait during soybean domestication. Thus, to test this speculation, we ordered 18 wild soybean (*G. soja*) and 23 cultivated soybean (*G. max*) accessions from the USDA which were used in the research of Sun et al. [9]. No mutations were found in either gene in the 18 wild soybean accessions. However, we observed SNPs in *GmHs1-1* simultaneously with *GmqHS1* in all 23 cultivated soybean accessions (Figure 1b). These results indicated that *GmHs1-1* and *GmqHS1* were selected together during soybean domestication, which might be the reason why functional *GmHs1-1* or *GmqHS1* only partially recovered the seed coat permeability phenotype in previous reported studies. Currently, the single-origin theory of the domestication of soybean appears to be widely accepted and also supported by marker analysis [19] and recent genome resequencing data [20,21]. The current understanding of the *GmHs1-1* and *GmqHS1* genes, together with advances for future research and utilization of the hard-seededness trait of soybean, was summarized recently [22].

During the process of soybean’s transition from wild to domesticated, the removal of hard-seededness characteristics affected the seed’s ability to absorb water and germinate. The classic imbibition process of wild soybean (*G. soja*) and cultivated soybean seeds (*G. max*) was described in this study (Figure 1c). Additionally, compared with wild soybean, the vigor and lifespan of cultivated soybean seeds after the breakdown of the impermeable seed coat are also affected. Based on previous reports of both hard and non-hard soybean seeds stored at room temperature (20 °C), it was found that non-hard seeds lost their vigor after approximately 2 years, while hard seeds still maintained a higher germination percentage of over 90% after 4 years [23]. As the main channel for germinating seeds to absorb water and exchange gases from the external environment, the seed coat and hilum are closely related to the maintenance of vigor and longer lifespan in hard soybean seeds. They hinder or restrict the influence of external water and gases on seeds, causing seeds to remain in a dormant or semi-dormant state for a long time, reducing the consumption of nutrients to the minimum level, thereby maintaining and extending seed vigor and lifespan. Overall, although the removal of hard-seededness characteristics in soybean is beneficial for seed germination, cultivation and production, it to some extent damages seed vigor and longevity under normal storage conditions. All these data indicate that both *Hs1-1* and *qHS1* are critical in controlling seed coat impermeability/hard-seededness. The simultaneous mutations of these two genes may have enabled artificial selection to break down hard-seededness during soybean domestication.

## 3. Materials and Methods

### 3.1. Plant Materials, Plant Growth and Growth Conditions

Wild soybean (*G. soja*) and cultivated soybean (*G. max*) accessions were requested from the USDA Soybean Germplasm Collection (Table 1). These populations were used for *GmHs1-1* and *GmqHS1* mutation variation analyses and association tests. Plants were grown at 22 °C day/20 °C night temperatures under a 16 h day/8 h night photoperiod in greenhouse conditions.

### 3.2. Seed Imbibition Phenotyping

Seeds of 18 wild soybean (*G. soja*) and 23 cultivated soybean (*G. max*) accessions were simultaneously harvested and used for seed coat impermeability tests. After the seeds were incubated in sterile water at room temperature for 2 h, the imbibed seeds were counted and the proportion of seeds with seed coat permeability was calculated. Cultivated soybean seeds showed imbibition, while wild soybean seeds did not. Additionally, the imbibition process of wild-type and mutant seeds was recorded by measuring the increased weight of imbibed seeds compared with their original dry weight at an interval of 40 min for 360 min.

### 3.3. DNA Isolation, PCR, Sequencing and Alignments

Genomic DNA was isolated from leaves of 4-week-old seedlings using a classical CTAB method (https://www.nature.com/articles/nprot.2006.384). PCR primers were designed to amplify the SNPs of the *Hs1-1* and *qHS1* genes (Table 2). The amplified fragment length was 190 bp for *qHS1* and 388 bp for *Hs1-1*. The PCR cycling conditions included an initial denaturation step at 95 °C for 5 min, followed by 35 cycles of denaturation at 95 °C for 15 s and annealing at 55 °C for 30 s. PCR products were directly sequenced, and alignment of the PCR product nucleotide sequences was carried out with DNASTAR Lasergene 12 (https://www.dnastar.com/software/).

## 4. Conclusions

All the above data indicate that the *Hs1-1* and *qHS1* genes are critical in controlling seed coat impermeability/hard-seededness. The simultaneous mutations of these two genes may have enabled artificial selection to break down hard-seededness during soybean domestication.

## Figures and Tables

**Table 1:** *G. soja* and *G. max* population samples from the USDA Soybean Germplasm Collection used for genotyping and phenotyping.

No.	Accession	Allele	Phenotype	Category	Province/State	Country
1	PI 483464A	*GmHs1-1 GmqHS1*	Hard	*Glycine soja*	Ningxia	China
2	PI 407301	*GmHs1-1 GmqHS1*	Hard	*Glycine soja*	Jiangsu	China
3	PI 407140	*GmHs1-1 GmqHS1*	Hard	*Glycine soja*	Kumamoto	Japan
4	PI 326582A	*GmHs1-1 GmqHS1*	Hard	*Glycine soja*	Primorye	Russia
5	PI 483465	*GmHs1-1 GmqHS1*	Hard	*Glycine soja*	Shaanxi	China
6	PI 464935	*GmHs1-1 GmqHS1*	Hard	*Glycine soja*	Jiangsu	China
7	PI 468400A	*GmHs1-1 GmqHS1*	Hard	*Glycine soja*	Ningxia	China
8	PI 468916	*GmHs1-1 GmqHS1*	Hard	*Glycine soja*	Liaoning	China
9	PI 339871A	*GmHs1-1 GmqHS1*	Hard	*Glycine soja*	Cheju	Korea
10	PI 458538	*GmHs1-1 GmqHS1*	Hard	*Glycine soja*	Heilongjiang	China
11	PI 597459D	*GmHs1-1 GmqHS1*	Hard	*Glycine soja*	Shandong	China
12	PI 393551	*GmHs1-1 GmqHS1*	Hard	*Glycine soja*	Taiwan	China
13	PI 597461A	*GmHs1-1 GmqHS1*	Hard	*Glycine soja*	Shandong	China
14	PI 407131	*GmHs1-1 GmqHS1*	Hard	*Glycine soja*	Kumamoto	Japan
15	PI 447004	*GmHs1-1 GmqHS1*	Hard	*Glycine soja*	Jilin	China
16	PI 562559	*GmHs1-1 GmqHS1*	Hard	*Glycine soja*	Cholla Puk	S. Korea
17	PI 366120	*GmHs1-1 GmqHS1*	Hard	*Glycine soja*	Akita	Japan
18	PI 407170	*GmHs1-1 GmqHS1*	Hard	*Glycine soja*	Kyonggi	S. Korea
19	PI 536637	*Gmhs1-1 Gmqhs1*	Permeable	*Glycine max*	South Carolina	USA
20	PI 548985	*Gmhs1-1 Gmqhs1*	Permeable	*Glycine max*	South Carolina	USA
21	PI 518664	*Gmhs1-1 Gmqhs1*	Permeable	*Glycine max*	Virginia	USA
22	PI 548604	*Gmhs1-1 Gmqhs1*	Permeable	*Glycine max*	Missouri	USA
23	PI 548520	*Gmhs1-1 Gmqhs1*	Permeable	*Glycine max*	Iowa	USA
24	PI 522236	*Gmhs1-1 Gmqhs1*	Permeable	*Glycine max*	Georgia	USA
25	PI 533655	*Gmhs1-1 Gmqhs1*	Permeable	*Glycine max*	Illinois	USA
26	PI 508266	*Gmhs1-1 Gmqhs1*	Permeable	*Glycine max*	North Carolina	USA
27	PI 515961	*Gmhs1-1 Gmqhs1*	Permeable	*Glycine max*	Kentucky	USA
28	PI 533602	*Gmhs1-1 Gmqhs1*	Permeable	*Glycine max*	Arkansas	USA
29	PI 548638	*Gmhs1-1 Gmqhs1*	Permeable	*Glycine max*	Ontario (Guelph)	Canada
30	PI 553047	*Gmhs1-1 Gmqhs1*	Permeable	*Glycine max*	Georgia	USA
31	PI 513382	*Gmhs1-1 Gmqhs1*	Permeable	*Glycine max*	Minnesota	USA
32	PI 548644	*Gmhs1-1 Gmqhs1*	Permeable	*Glycine max*	Ontario (Guelph)	Canada
33	PI 536635	*Gmhs1-1 Gmqhs1*	Permeable	*Glycine max*	Ohio	USA
34	PI 548643	*Gmhs1-1 Gmqhs1*	Permeable	*Glycine max*	Ontario (Ottawa)	Canada
35	PI 508083	*Gmhs1-1 Gmqhs1*	Permeable	*Glycine max*	Minnesota	USA
36	PI 525453	*Gmhs1-1 Gmqhs1*	Permeable	*Glycine max*	Iowa	USA
37	PI 548634	*Gmhs1-1 Gmqhs1*	Permeable	*Glycine max*	Ohio	USA
38	PI 542403	*Gmhs1-1 Gmqhs1*	Permeable	*Glycine max*	Minnesota	USA
39	PI 540552	*Gmhs1-1 Gmqhs1*	Permeable	*Glycine max*	Ohio	USA
40	PI 556511	*Gmhs1-1 Gmqhs1*	Permeable	*Glycine max*	Michigan	USA
41	PI 548512	*Gmhs1-1 Gmqhs1*	Permeable	*Glycine max*	Indiana	USA

**Table 2:** Primers used in this study.

Source of Gene Sequence	Primer Name	Primer Sequence	Application
Glyma.02G269400	*GmqHS1* F	GCTACTATGCGTCGGTGAGT	For cloning of the SOYBEAN *GmqHS1* and SNP sequencing
Glyma.02G269400	*GmqHS1* R	GAGACCATGAGTTGAAGGCCA	For cloning of the SOYBEAN *GmqHS1* and SNP sequencing
Glyma.02G269500	*GmHs1-1* F1	TGGGCTTTCATTAGGTCAGACAAAC	For cloning of the SOYBEAN *GmHs1-1* and SNP sequencing
Glyma.02G269500	*GmHs1-1* R1	TCGAAGGAGTGACATCCAAGGTAA	For cloning of the SOYBEAN *GmHs1-1* and SNP sequencing

## Data Availability

Data is contained within the article.
